# Microfocused Ultrasound With Visualization Induces Remodeling of Collagen and Elastin Within the Skin

**DOI:** 10.1111/jocd.16638

**Published:** 2024-11-15

**Authors:** Kay Marquardt, Christian Hartmann, Flora Wegener, Je‐Young Park, Douglas Halbert, Stephen Hsu, Thomas Hengl

**Affiliations:** ^1^ R&D Skin Lab & Nonclinical Science Department Merz Aesthetics GmbH Frankfurt am Main Germany; ^2^ Apkoo‐Jung Oracle Dermatology Center Seoul Republic of Korea; ^3^ Merz North America, Inc. Raleigh North Carolina USA

**Keywords:** energy‐based device (EBD), extracellular matrix (ECM), healing process, microfocused ultrasound, regeneration, thermal coagulation point (TCP)

## Abstract

**Purpose:**

Microfocused ultrasound with real‐time visualization (MFU‐V) is often used for noninvasive skin lifting, by precisely targeting dermal and subcutaneous tissues to create thermal coagulation points (TCPs). These TCPs denature collagen and initiate a transient inflammatory response, ultimately attracting dermal fibroblasts and inducing efficient neocollagenesis and extracellular matrix (ECM) remodeling, yielding to MFU‐V's desired skin‐lifting effects. The current study investigates MFU‐V's underlying mode of action based on the histological progression of TCPs in the skin, providing new insight into the technology's regenerative effect.

**Methods:**

Following standard triple‐depth MFU‐V treatment, in vivo skin samples were assessed using histology and immunohistochemistry to evaluate TCPs, heat shock protein (HSP47), and elastin expression in fibroblasts.

**Results:**

MFU‐V treatment induced elongated, flame‐like TCPs with denatured collagen at focal depths of 1.5, 3.0, and 4.5 mm within the skin—each corresponding to its respective transducer depth. Time‐dependent progression of TCPs showed significantly increased scores of fibroblasts and mature collagen along with recruitment of HSP47‐positive fibroblasts to TCP areas on Day 90. Collagen formation and later maturation were visualized. Newly synthesized elastin significantly increased in the TCP area on Day 90 compared to Day 14.

**Conclusion:**

This work provides histological evidence of stimulation and regeneration of newly synthesized elastin fibers after TCP induction. MFU‐V‐generated TCPs triggered the body's own healing cascade of collagen denaturation, transient inflammation, proliferation, and tissue remodeling, resulting in attraction of HSP47‐positive fibroblasts to the TCP sites, and new collagen and elastin fiber regeneration by fibroblasts. Besides the well‐described neocollagenesis, this study demonstrates that MFU‐V treatment induces elastin neogenesis that may result not only in skin lifting but also in improved skin elasticity, providing an overall regenerative effect.

## 1 Introduction

Elective, nonsurgical, cosmetic procedures have become increasingly popular (> 7 million procedures in 2022 in the United States alone) over the last decade. Microfocused ultrasound (MFU) instruments are noninvasive devices capable of heating tissue to greater than 60°C, producing distinct thermal coagulation points (TCPs) by precisely targeting dermal and subcutaneous tissue at specific focal depths, without affecting the overlying papillary dermal and epidermal layers [1, 2]. This MFU procedure provides patients with skin lifting and no downtime due to its targeted treatment as compared to traditional facelift surgeries [3, 4]. The aesthetic success of MFU treatment has been demonstrated in numerous studies [5, 6, 7, 8, 9, 10, 11, 12]. Currently, the Ulthera System, with its DeepSEE transducers (Ulthera, Merz North America, Inc., Raleigh, NC, USA), is the only FDA‐cleared noninvasive skin‐lifting treatment that employs MFU with real‐time visualization (MFU‐V) to lift the skin of the eyebrow, neck, and submentum as well as to improve lines and wrinkles of the décolleté [13].

In contrast to high‐powered ultrasound devices, Ulthera's proposed mechanism involves the precise delivery of ultrasound energy to targeted depths; superficial dermis at 1.5 mm, deep dermis at 3.0 mm, and superficial musculoaponeurotic system (SMAS)/platysma at 4.5 mm, thereby generating controlled TCPs and denaturation of ECM protein within milliseconds [14]. This process leads to further contraction and coagulation of collagen and other ECM proteins, initiates the body's own healing response, and prompts the production of new ECM proteins like collagen and elastin over time [15, 16].

Given the abundant clinical success of MFU‐V treatment, information related to the underlying physiological and molecular pathways of activation via TCPs and histological evaluation of MFU‐V‐treated skin remains to be characterized [8, 9, 10].

To provide deeper insights into the mode of action of MFU‐V, a time‐resolved histological evaluation of treated skin was performed. The heat shock protein 47 (HSP47) was used to track the progression of neocollagenesis since HSP47 is expressed by ECM‐producing fibroblasts and is known as a collagen‐specific chaperone that plays crucial role in the synthesis, maturation, and stability of collagen fibers [17]. Neogenesis of elastin fibers posttreatment was also analyzed; restoration of ECM proteins is an important aspect of the natural healing process at thermal coagulation sites and elastin is essential for restoring the skin's structural integrity and function [18].

The current work indicates that following MFU‐V treatment, a cascade of physiological and molecular changes is triggered. These include general ECM denaturation and healing progression, subsequently resulting in recruitment of new fibroblasts inducing neocollagenesis and neoelastogenesis. Overall, this study evaluates a potentially novel and previously unreported molecular mechanism of action of how MFU‐V improves skin lifting and tightening, and ultimately skin regeneration, in patients seeking noninvasive treatment.

## 2 Material and Methods

### 2.1 In Vivo Skin Treatment

Skin sites (Yucatan, Miniature Swine) were treated with MFU‐V energy device (Ulthera, Merz North America, Inc., Raleigh, NC USA) at focal depths of 1.5, 3.0, and 4.5 mm. A total number of 20 sites with a dimension of 25 × 25 mm were treated in a triple‐depth approach using all transducers (10 MHz–1.5 mm, 7 MHz–3.0 mm, and 4 MHz–4.5 mm) with energy level settings at 2 (0.18, 0.3, and 0.9 J) or 4 (0.25, 0.45, and 1.2 J). The treatment sites were excised and fixed in 10% neutral buffered formalin (NBF, Sigma Aldrich, HT501128) on Day 0 (*n* = 4), Day 14 (*n* = 8), and Day 90 (*n* = 8) posttreatment. All experiments followed the Guide for the Care and Use of Laboratory Animals (Eighth Edition, 2011; ILARCLS, National Research Council, Washington, DC) and were approved by an Institutional Animal Care and Use Committee (IACUC).

### 2.2 Histological Assessment

NBF‐fixed skin samples were dehydrated in ethanol solutions of increasing concentration, cleared in xylene (Sigma Aldrich, #214736), and embedded in paraffin wax (Merck, #107151). The paraffin blocks were then sectioned at 4 ± 1 μm using a microtome (4 slides per treatment site; total: 80 sections). For histologic examination, the sections were stained with safranin–hematoxylin–eosin (SHE) (Milipore, #M380112, Thermofisher, #047223.14, #152885000), and a ACVP‐certified histopathologist performed individual semiquantitative histological assessment scoring for the following parameters: collagen maturity, epithelization, edema, hemorrhage/hemosiderin, fibroplasia, cell/tissue degeneration, hyalinized collagen, serocellular crust, chronic inflammation at subcutis, overall inflammation, polymorphonuclear cells, lymphocytes, plasma cells, macrophages, giant cells, necrosis, and fibrosis. The scoring was performed as per rating scheme (Table 1). Findings with scores > 0 were considered for further data analysis.

**TABLE 1:** Semiquantitative histological assessment scoring.

Score	Findings
0	Not present
1	Minimal: present, but slight feature
2	Mild: notable feature not effacing or disrupting preexisting tissue elements or limited to a small tissue area
3	Moderate: prominent or relatively widespread feature that does not disrupt tissue architecture and is not overwhelming
4	Marked/severe: overwhelming feature that is very widespread or complete, and/or effaces or disrupts tissue architecture

Abbreviations: EBD, energy‐based device; ECM, extracellular matrix; HSP47, heat shock protein 47; IHC, immunohistochemistry; MFU, microfocused ultrasound; MFU‐V, microfocused ultrasound with visualization; SHE, safranin–hematoxylin–eosin; SMAS, superficial musculoaponeurotic system; TCP, thermal coagulation point; TGF‐β, transforming growth factor beta.

### 2.3 Immunohistochemistry (IHC)

The skin section slides for Day 14 and Day 90 with a histological assessment score > 0 were prepared for IHC as follows:

Dewaxing of paraffin‐embedded samples was performed using Bond Dewax Solution (Biosystems #AR9222) followed by sequential ethanol rinsing steps (EtOH 100%) and washing steps using Bond Wash Solution (Biosystems, #AR9590). Antigen Retrieval was performed using Proteinase K (Merck, #21627) in a 1:10 dilution for 30 min at room temperature (RT). Primary antibodies against HSP47 (Enzo, #ADI‐SPA‐470) in a dilution of 1:400 for 1 h at RT, and elastin (Abcam, #ab23747) in a dilution of 1:1000 for 1 h an RT were used. Counterstaining was performed with Bond Polymer Refine Detection Kit (Biosystems, #DS9800). For the staining procedure, an automatic IHC stainer (Leica Bond IIITM) was used. The slides were imaged using 20× objective on an Olympus Slideview VS200 slide scanner with a VS‐264C camera. Semiquantitative histomorphometry was performed for selective samples using the bioimage analysis software QuPath (version 0.3.2).

The slides were manually annotated for TCPs and were expanded 300 μm with the original inner TCP annotation subtracted from the expanded annotation area. Within the TCP annotation and their expanded area annotation, a watershed algorithm plugin for cell detection and IHC‐positive cell detection were conducted for HSP47. IHC‐positive elastin fibers were manually annotated for pixel/area within the TCPs.

### 2.4 Data Analysis and Statistical Quantification

Statistical analyses of IHC slides were performed for 316 TCPs (Day 14, *n* = 156; Day 90, *n* = 160) and conducted in GraphPad Prism 9 (GraphPad Software, San Diego, USA). One‐way ANOVA with post hoc Šídák's multiple comparisons tests were performed, and *p*‐values < 0.05 were considered statistically significant.

## 3 Results

### 3.1 MFU‐V–Induced TCPs in the Skin

**FIGURE 1:**
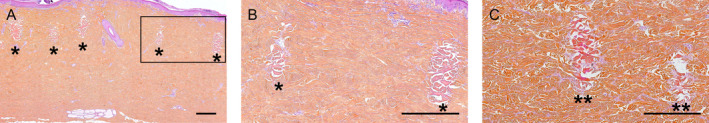
MFU‐V induced TCPs in the dermal layer of skin: Skin sections displaying small, inverted cone‐like multifoci with collagen denaturation in the dermis at regular intervals (A). Detailed TCPs showing focal collagen denaturation, characterized by loss of fine fibrillar structure at the different depths of 1.5 mm (*) (B) and 3.0 mm (**) (C). Images represent sections at 14 days posttreatment. Scale bars indicate 500 μm.

To understand the mechanism of action of the MFU‐V energy device, the skin was analyzed for focal TCPs using histologic evaluation on Day 14 and Day 90 posttreatment using SHE staining. The staining revealed no superficial layer ablation or other side effects in the epidermal layer as indicated in Figure 1. On Day 14, the TPCs looked elliptical‐to‐inverted cone‐like and approximately 500 × 200 μm in size with vertical long axis perpendicular to the skin surface. TCPs were characterized by focal hyalinized collagen with loss of fine fibrillar structures and absence of nuclei and the focal depth of the TCPs correlated with the respective transducers used (Figure 1).

### 3.2 Time‐Dependent Histological Changes in the TPCs Favoring Remodeling of Collagen

Overall, histological assessment gave a score of 0 for most parameters, underlining the general safety of MFU‐V treatment and effectiveness in eliminating the respective parameters from further consideration.

**FIGURE 2:**
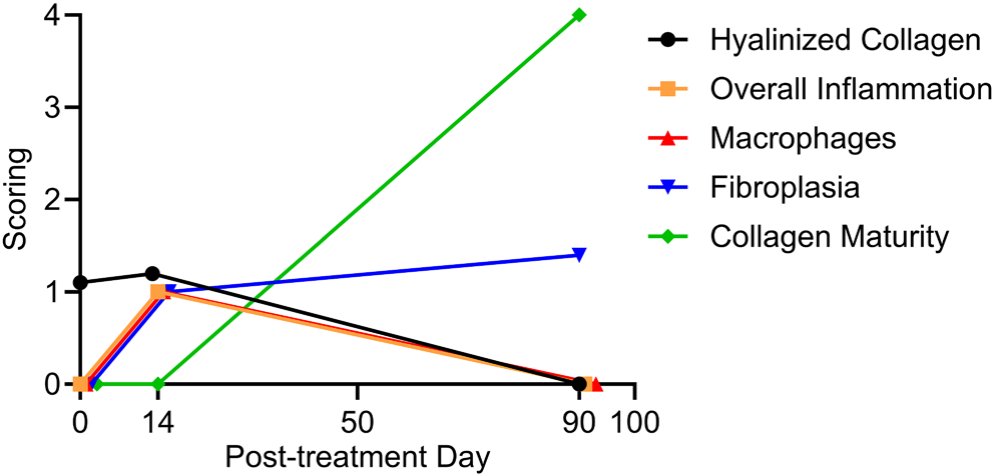
Time‐dependent histological progression of TCPs after MFU‐V treatment. Fibroblast consistently started to grow around Day 14 through Day 90 along with collagen maturity. Transient inflammation on Day 14 together with increased macrophage levels is present, but strongly decreases till Day 90.

The histological assessment portrayed time‐dependent changes in the TCP area within the skin (Figure 2) where TCP foci were highly visible on Day 0, with eosinophil structures and denatured collagen (stated as hyalinized collagen); after Day 14, cell infiltration started to accumulate at the TCP edges mainly consisting of fibroblasts (stated as fibroplasia) and few macrophages. Macrophages, T cells, and rare number of giant cells were scored as a sign of inflammation on Day 14. These signs of inflammation completely resolved on Day 90 followed by the accumulation of fibroblasts at the edges as well as in the internal spaces of the TCPs. Hyalinized collagen was no longer visible on Day 90 and was being remodeled into new collagen deposition with increased cellularity compared to adjacent areas, resulting in an arrangement of new tissue with new collagen fibers.

### 3.3 Increase in HSP47 Expressing Fibroblasts at the TCP Area

**FIGURE 3:**
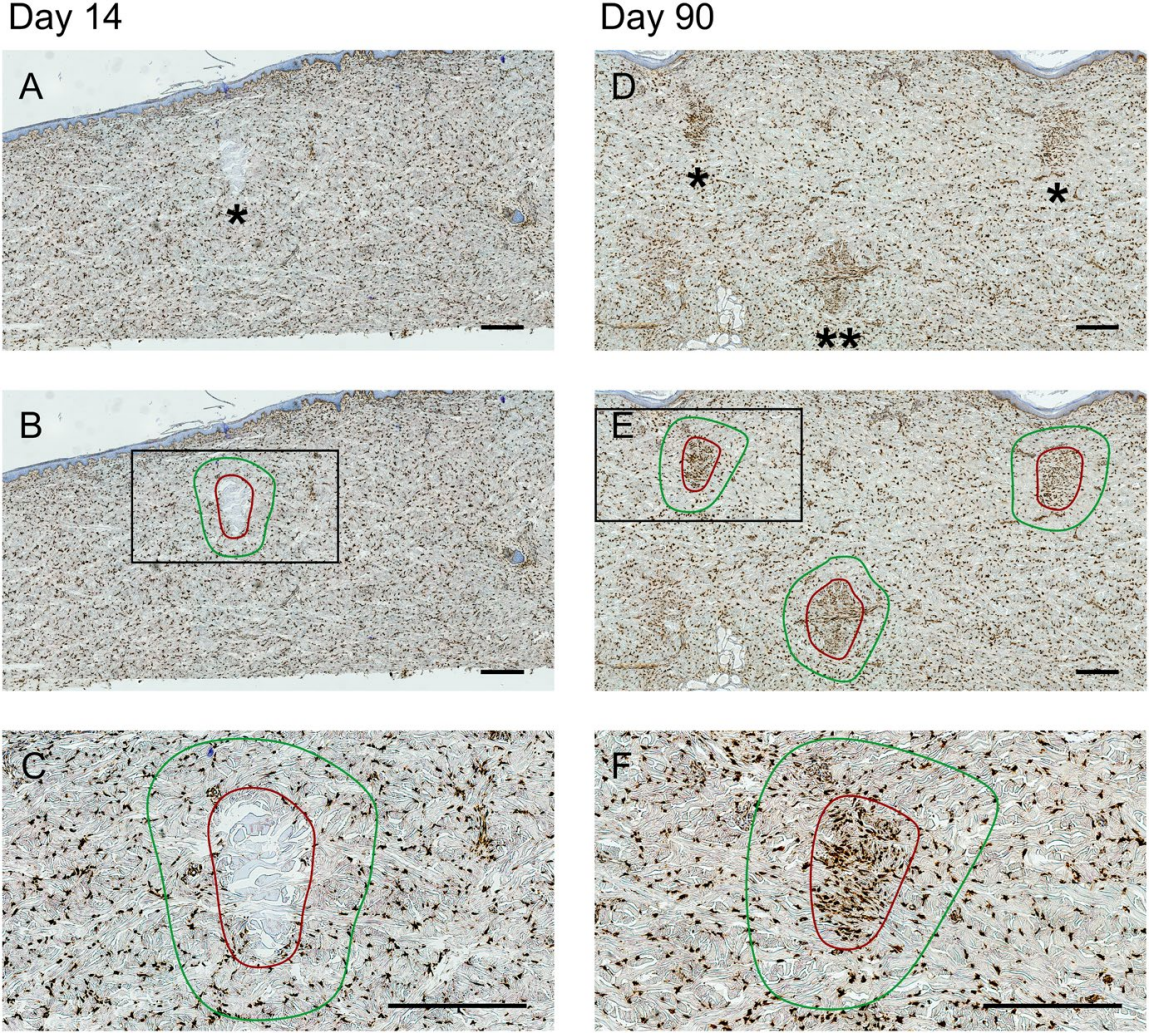
Visualization of HSP47‐positive fibroblasts within and in proximity to TCPs on Day 14 and Day 90 post–MFU‐V treatment. Overview image with asterisk indicating TCPs at the depth of 1.5 mm (*) and 3.0 mm (**) on Days 14 (A) and 90 (D). Red encircled area indicates the direct TCP area, green encircled area indicates the 300 μm^2^ expansion area around the TCP on Days 14 (B) and 90 (E). HSP47‐positive fibroblasts were analyzed within TCPs (red circle) and in an expansion area of 300 μm^2^ surrounding the TCPs (green circle) on Days 14 (C) and 90 (F). Scale bars indicate 500 μm.

**FIGURE 4:**
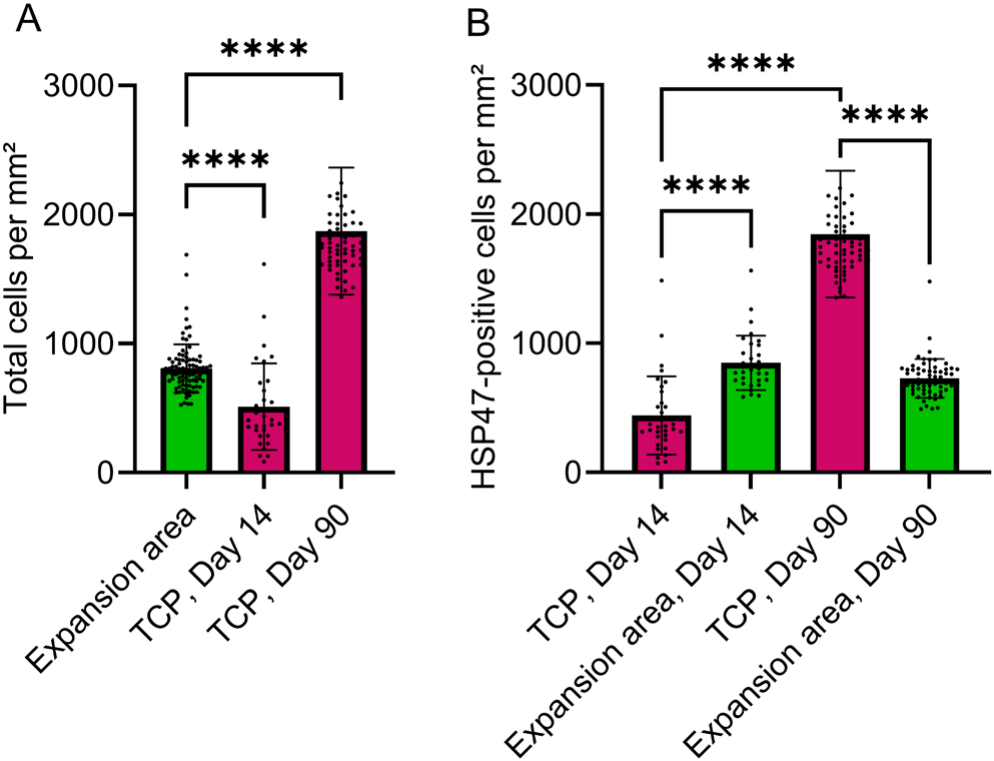
Quantification of HSP47‐positive fibroblasts in TCPs and in the respective expansion area after Day 14 and Day 90 post–MFU‐V treatment. Total cell number per mm^2^ increased in TCPs after Day 90 compared to cell number on Day 14 and compared to the pooled expansion area on Day 14 and Day 90 (A, ANOVA Šídák's multiple comparison ****p < 0.0001). Number of HSP47‐positive fibroblasts is reduced in the TCP area compared to the expansion area on Day 14 but increased significantly on Day 90 compared to its respective expansion area (B, ANOVA Šídák's multiple comparison ****p < 0.0001). Bars represent mean, and error bars represent standard error of the mean.

Histological analyses of TCPs on a cellular level were performed. After MFU‐V treatment, total cell numbers in the TCP significantly increased on Day 90 (mean ± SD: 1817 ± 488 per mm^2^) compared to Day 14 and compared to mean value of the expansion area of the TCP on Day 14 and Day 90 (512 ± 330 per mm^2^ vs. 807 ± 185 per mm^2^, *p* < 0.0001, respectively) (Figures 3C,F and 4A).

Further analysis of the cell population was performed using HSP47‐specific antibody staining to identify HSP47‐expressing fibroblasts. HSP47 is an established marker for collagen‐producing fibroblasts known to be crucial for collagen development and stability. On Day 14, a greater number of HSP47‐positive fibroblasts were recruited in the expansion area compared to the TCP (849 ± 206 vs. 440 ± 298 cells per mm^2^, *p* < 0.0001) (Figure 3C,F; Figure 4B). In the expansion area, 84.8% ± 9.5% of the cells were HSP47‐positive, whereas immune infiltrate, predominantly macrophages, were present at the border proximity (Figure 3C).

In contrast, on Day 90, the TCPs were highly infiltrated with elongated and/or spindle‐shaped fibroblastic‐appearing cells (Figure 3F), and HSP47‐positive cell numbers were significantly increased in the TCPs compared to the expansion area (1846 ± 485 vs. 729 ± 148 cells per mm^2^, *p* < 0.0001) (Figure 4B). Considering the total cell population within the TCPs, 98.6% ± 1.3% of the cells were HSP47 positive on Day 90, indicating initiation of collagen remodeling by fibroblasts.

### 3.4 Stimulation of Elastin Neogenesis in the TCPs

**FIGURE 5:**
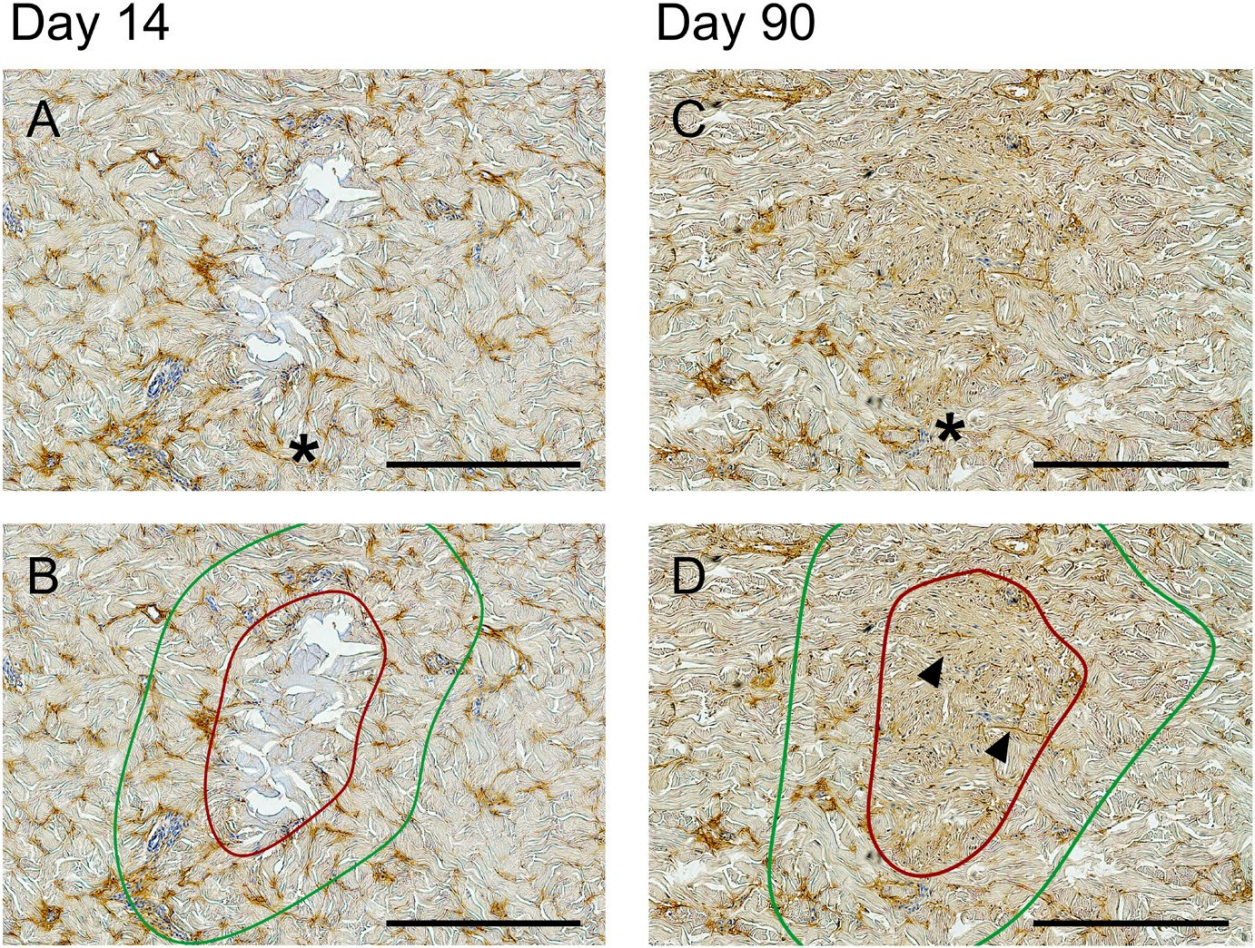
Visualization of elastic fibers and elastin in proximity and within the TCPs on Day 14 and Day 90 post–MFU‐V treatment. Overview image with asterisk indicating TCPs at a depth of 1.5 mm on Days 14 (A) and 90 (C) and encircling of the direct TCP area (indicated by the red circle) and the expansion area (indicated by the green circle) on Days 14 (B) and 90 (D). Arrowheads show newly formed elastin fibers. Scale bars indicate 500 μm.

**FIGURE 6:**
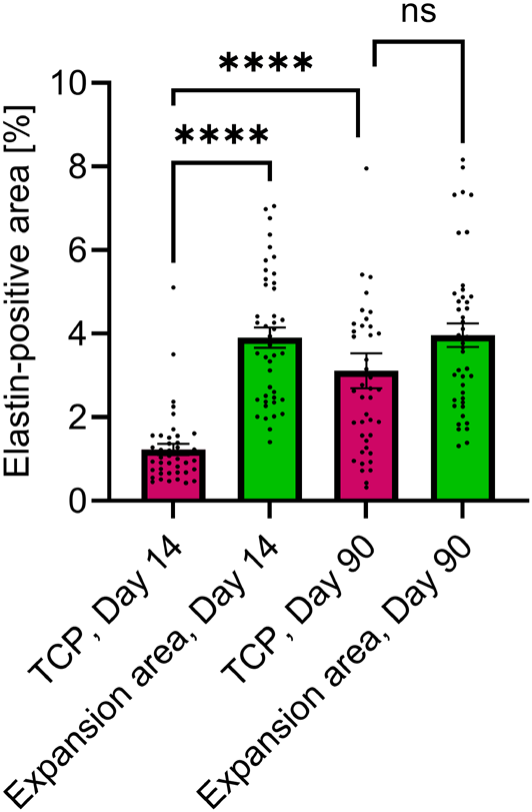
Semiquantification of elastin‐positive area in proximity and within the TCPs on Day 14 and Day 90 post–MFU‐V treatment. The elastin‐positive area within the TCP is significantly increased on Day 90 compared to Day 14 (****p < 0.0001) but remains similar to its respective expansion area (ANOVA Šídák's multiple comparison). Bars represent mean, and error bars represent standard error of the mean.

In addition to neocollagenesis, the potential effect of MFU‐V treatment on elastin generation outside and inside the TCP was assessed. On Day 14, 3.9% ± 1.5% of the expansion area was elastin positive compared to 1.2% ± 0.8% within the TCP (*p* < 0.0001) (Figures 5A,B and 6). In contrast, on Day 90, elastin fibers were found inside the TCPs as indicated in Figure 5C,D. The elastin‐positive area was similar in the expansion area and the TCPs (3.9% ± 1.8% vs. 3.1% ± 2.7%, *p* = ns, respectively) (Figure 6). The direct comparison of the elastin‐positive area within the TCPs on Day 14 and Day 90 indicated a twofold increase in elastin (1.2% ± 0.8% vs. 3.1% ± 2.7%, *p* < 0.0001) providing evidence for neogenesis of elastic fibers after MFU‐V treatment (Figure 6).

## 4 Discussion

Ultherapy, the only FDA‐cleared treatment using MFU‐V, successfully induced distinct inverted cone‐like TCPs at specific depths ranging from superficial to deep dermis without affecting the structure and integrity of the epidermis, highlighting the noninvasive character of the MFU‐V treatment. Noninvasive MFU devices have been used in the past for skin lifting, but Ultherapy goes beyond by visualizing focal depths during the treatment [19, 20]. Several studies have shown that MFU devices induce thermal injury zones and lead to long‐lasting skin‐lifting effects; however, the mechanism of action at the molecular level remained unknown [21, 22].

**FIGURE 7:**
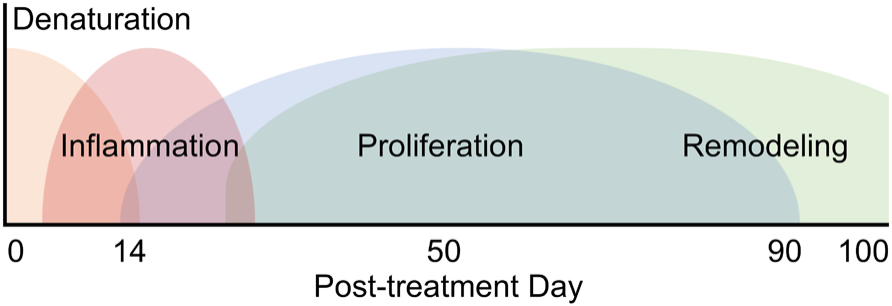
Proposed stages of histological response to MFU‐V based on the current results of this study.

The data from the current study reveal a histological time progression involving transient inflammation, proliferation, and remodeling consistent with a natural healing response (Figure 7), without evidence of superficial epidermal damage, suggesting that ECM remodeling can be achieved without the drawbacks of invasive, wound‐inducing procedures [23].

Based on the histological analyses, we found that MFU‐V treatment activated molecular pathways that recruited inflammatory cells to the TCPs. Inflammation is a sign of the body's own healing process as tissue repairs itself by forming new tissue and activating the immune system [24]. Macrophage cells and giant cells were observed at the TCP sites around Day 14, but decreased in number over time as fibroblasts became the main cell population by Day 90, presenting a successful mechanistic transition from inflammation to proliferation and remodeling [25]. This is consistent with the general understanding that multiple cell types are involved during the healing process, with fibroblasts as the key cell type for later connective tissue healing and restoration [26]. During the late stages of healing progression, activated fibroblasts maintain the cross‐linkage and turnover of deposited ECM, including the partial transition of Collagen III to Collagen I fibers and the maturation, strengthening, and stiffening of the ECM [18, 26]. The histological analysis of the TCPs indicated the recruitment of HSP47‐positive fibroblasts to the TCP area following the generation of newly synthesized ECM fibers—mainly consisting of collagen and elastin—suggesting a natural remodeling and restoration of the coagulated ECM found at the early time points posttreatment.

In support of proper folding and later maturation of collagen is the HSP47 chaperone which has been also described as the rate‐limiting factor for Collagen I synthesis [17, 27]. The accumulation of HSP47‐positive fibroblasts in TCPs may be explained by the need to form new collagen and elastin fibers as part of the healing process. These results are in accordance with the study by Sasaki et al., which found induced neosynthesis of Collagen I and III analyzed 4 weeks after MFU treatment [28]. Activation of transforming growth factor β (TGF‐β) has been suggested as a starting point for the pathways underlying neocollagenesis via HSP47‐induced stimulation [29]. The initial collagen contraction along with the described natural healing response may explain the clinical effects of lifting and tightening [16, 30, 31]. The presented remodeling effect in the skin can be correlated with clinical Cutometer measurements, where the net and gross elasticity of the skin was improved after 12 weeks, suggesting that MFU‐V is a suitable treatment for patients with loss of elasticity and contour in the areas such as the face [10]. Other relevant areas may include soft tissue with age‐related skin laxity in the region of the chest, buttocks, or arms [36, 37]. The remodeling effect may also explain the improvement in clinical skin quality parameters such as minimization of facial pores and improvement in skin texture [32]. Neocollagenesis and volume restoration induced by energy‐based devices can be supplemented with fillers to achieve optimal clinical results in a minimally invasive way [33, 34, 35]. The current study provides information on the TCP remodeling progression, which may help to determine a good time point to combine such treatments.

In addition to the ECM component collagen, elastin was also affected and coagulated by the MFU‐V treatment. Over time, elastin staining increased significantly and reached similar levels to that of the surrounding tissue, providing evidence for neogenesis of elastin fibers after MFU‐V treatment. Clinical translation of the molecular effects of MFU‐V treatment includes increased tensile strength of the skin (collagen) and greater elasticity (elastin). Altogether, MFU‐V treatment causes activation of neocollagenesis and neoelastogenesis molecular pathways at TCPs, leading to a shrinking and tightening of the skin facilitated by the body's own capacity to remodel and restore connective tissue.

There are a few limitations to the current analysis. The characterization of focal TCPs, analysis of HSP47‐recruited fibroblasts, and elastin neogenesis histology were performed using processed ex vivo skin samples from miniature swine. Processing of skin tissue resulted in tissue shrinkage, which alter the dimension of the skin. Porcine skin is similar to human skin in many ways, such as skin layer thickness, hair and gland density, and healing process. However, it lacks the SMAS with collagenous structures, a target site at the 4.5 mm depth, limiting our analysis to the two remaining depths of 1.5 and 3.0 mm. While it may be possible to extrapolate the response of collagenous structures within the dermis to collagenous structures in general, the interaction of the SMAS with the overlying skin cannot be explored. Additional in vitro research is needed to support these findings, along with further clinical research to confirm the proposed mechanism in humans.

## 5 Conclusion

In this study, Ultherapy—which utilizes MFU‐V–based transducers—successfully created TCPs at specific depths in the skin without affecting the epidermal layer. The TCPs triggered a cascade of the body's own healing mechanisms. Inflammation was transient at these sites, while the proliferation and remodeling stages lasted longer, involving recruitment of HSP47‐positive fibroblasts to the TCP area for production of new collagen and elastin. In addition to previously described neocollagenesis, this study demonstrates that MFU‐V treatment also induces elastin neogenesis, which may result in skin lifting and improved elasticity, providing an overall regenerative effect. Together, these results provide time‐resolved insights into the remodeling of collagen and elastin in the skin by MFU‐V, adding new insights into the underlying cellular responses of this treatment.

## Author Contributions

K.M., F.W., and T.H. performed the research. K.M., F.W., D.H., S.H., and T.H. designed the research study. K.M., C.H., and F.W. analyzed the data. K.M., C.H., F.W., and J.‐Y.P. wrote and reviewed the paper. All authors have read and approved the final manuscript.

## Conflicts of Interest

K. Marquardt, C. Hartmann, F. Wegener, and T. Hengl were employees of Merz Aesthetics GmbH. D. Halbert and S. Hsu were employees of Merz North America, Inc. J.‐Y. Park has no financial interest to disclose in relation to the content of the article. All authors contributed to the development and review of this work, agreed with the content, and report no conflicts of interest in this work.

## Data Availability

The data that support the findings of this study are available on request from the corresponding author. The data are not publicly available due to privacy or ethical restrictions.

## References

[1] S. G. Fabi , “Noninvasive Skin Tightening: Focus on New Ultrasound Techniques,” Clinical, Cosmetic and Investigational Dermatology 8 (2015): 47–52, 10.2147/CCID.S69118. 25709486 PMC4327394

[2] R. E. Gliklich , W. M. White , M. H. Slayton , P. G. Barthe , and I. R. S. Makin , “Clinical Pilot Study of Intense Ultrasound Therapy to Deep Dermal Facial Skin and Subcutaneous Tissues,” Archives of Facial Plastic Surgery 9, no. 2 (2007): 88–95, 10.1001/archfaci.9.2.88. 17372061

[3] M. Alam , L. E. White , N. Martin , J. Witherspoon , S. Yoo , and D. P. West , “Ultrasound Tightening of Facial and Neck Skin: A Rater‐Blinded Prospective Cohort Study,” Journal of the American Academy of Dermatology 62, no. 2 (2010): 262–269, 10.1016/j.jaad.2009.06.0394. 20115948

[4] H. S. Lee , W. S. Jang , Y. J. Cha , et al., “Multiple Pass Ultrasound Tightening of Skin Laxity of the Lower Face and Neck,” Dermatologic Surgery 38, no. 1 (2012): 20–27, 10.1111/j.1524-4725.2011.02158.x. 22092848

[5] M. Contini , M. H. J. Hollander , A. Vissink , R. H. Schepers , J. Jansma , and J. Schortinghuis , “A Systematic Review of the Efficacy of Microfocused Ultrasound for Facial Skin Tightening,” International Journal of Environmental Research and Public Health 20, no. 2 (2023): 1522, 10.3390/ijerph20021522. 36674277 PMC9861614

[6] M. K. Dobke , T. Hitchcock , L. Misell , and G. H. Sasaki , “Tissue Restructuring by Energy‐Based Surgical Tools,” Clinics in Plastic Surgery 39, no. 4 (2012): 399–408, 10.1016/j.cps.2012.07.008. 23036290

[7] S. G. Fabi , A. Massaki , S. Eimpunth , J. Pogoda , and M. P. Goldman , “Evaluation of Microfocused Ultrasound With Visualization for Lifting, Tightening, and Wrinkle Reduction of the Décolletage,” Journal of the American Academy of Dermatology 69, no. 6 (2013): 965–971, 10.1016/j.jaad.2013.06.045. 24054759

[8] S. G. Fabi , J. Joseph , J. Sevi , J. B. Green , and J. D. Peterson , “Optimizing Patient Outcomes by Customizing Treatment With Microfocused Ultrasound With Visualization: Gold Standard Consensus Guidelines From an Expert Panel,” Journal of Drugs in Dermatology 18, no. 5 (2019): 426–432. 31141851

[9] T. M. Hitchcock and M. K. Dobke , “Review of the Safety Profile for Microfocused Ultrasound With Visualization,” Journal of Cosmetic Dermatology 13, no. 4 (2014): 329–335, 10.1111/jocd.12111. 25399626

[10] M. Kerscher , A. T. Nurrisyanti , C. Eiben‐Nielson , S. Hartmann , and J. Lambert‐Baumann , “Skin Physiology and Safety of Microfocused Ultrasound With Visualization for Improving Skin Laxity,” Clinical, Cosmetic and Investigational Dermatology 12 (2019): 71–79, 10.2147/CCID.S188586. 30666145 PMC6336023

[11] G. Oni , R. Hoxworth , S. Teotia , S. Brown , and J. M. Kenkel , “Evaluation of a Microfocused Ultrasound System for Improving Skin Laxity and Tightening in the Lower Face,” Aesthetic Surgery Journal 34, no. 7 (2014): 1099–1110, 10.1177/1090820X14541956. 24990884

[12] D. H. Suh , M. K. Shin , S. J. Lee , et al., “Intense Focused Ultrasound Tightening in Asian Skin: Clinical and Pathologic Results,” Dermatologic Surgery 37, no. 11 (2011): 1595–1602, 10.1111/j.1524-4725.2011.02094.x. 21806707

[13] Ulthera® System (Ultherapy®) Instruction for Use (Raleigh, NC: Ulthera, Inc, 2024), https://ultherapy.com/ifu.

[14] T. J. Dubinsky , C. Cuevas , M. K. Dighe , O. Kolokythas , and J. H. Hwang , “High‐Intensity Focused Ultrasound: Current Potential and Oncologic Applications,” American Journal of Roentgenology 190, no. 1 (2008): 191–199, 10.2214/AJR.07.2671. 18094311

[15] K. Hayashi , G. Thabit, III , K. L. Massa , et al., “The Effect of Thermal Heating on the Length and Histologic Properties of the Glenohumeral Joint Capsule,” American Journal of Sports Medicine 25, no. 1 (1997): 107–112, 10.1177/036354659702500121. 9006703

[16] D. H. Suh , J. H. Choi , S. J. Lee , K. H. Jeong , K. Y. Song , and M. K. Shin , “Comparative Histometric Analysis of the Effects of High‐Intensity Focused Ultrasound and Radiofrequency on Skin,” Journal of Cosmetic and Laser Therapy 17, no. 5 (2015): 230–236, 10.3109/14764172.2015.1022189. 25723905

[17] Y. Ishida and K. Nagata , “Hsp47 as a Collagen‐Specific Molecular Chaperone,” Methods in Enzymology 499 (2011): 167–182, 10.1016/B978-0-12-386471-0.00009-2. 21683254

[18] B. Hinz , “Formation and Function of the Myofibroblast During Tissue Repair,” Journal of Investigative Dermatology 127, no. 3 (2007): 526–537, 10.1038/sj.jid.5700613. 17299435

[19] M. Alhaddad , D. C. Wu , J. Bolton , et al., “A Randomized, Split‐Face, Evaluator‐Blind Clinical Trial Comparing Monopolar Radiofrequency Versus Microfocused Ultrasound With Visualization for Lifting and Tightening of the Face and Upper Neck,” Dermatologic Surgery 45, no. 1 (2019): 131–139, 10.1097/DSS.0000000000001653. 30531187

[20] G. Casabona , K. Kaye , S. Cotofana , K. Davidovic , M. Alfertshofer , and L. Freytag , “Histological Effects of a Combined Collagen Stimulation Procedure Consisting of Microfocused Ultrasound, Soft Tissue Filler, and Ca‐HA Injections,” Journal of Cosmetic Dermatology 22, no. 6 (2023): 1724–1730, 10.1111/jocd.15770. 37073423

[21] W. M. White , I. R. Makin , P. G. Barthe , et al., “Selective Creation of Thermal Injury Zones in the Superficial Musculoaponeurotic System Using Intense Ultrasound Therapy: A New Target for Noninvasive Facial Rejuvenation,” Archives of Facial Plastic Surgery 9, no. 1 (2007): 22–29, 10.1001/archfaci.9.1.22. 17224484

[22] S. Oh , D. Y. Rhee , S. Batsukh , K. H. Son , and K. Byun , “High‐Intensity Focused Ultrasound Increases Collagen and Elastin Fiber Synthesis by Modulating Caveolin‐1 in Aging Skin,” Cells 12, no. 18 (2023): 2275, 10.3390/cells12182275. 37759497 PMC10527789

[23] T. J. Shaw and P. Martin , “Wound Repair at a Glance,” Journal of Cell Science 122, no. Pt 18 (2009): 3209–3213, 10.1242/jcs.031187. 19726630 PMC2736861

[24] R. I. Korelo , M. Kryczyk , C. Garcia , K. Naliwaiko , and L. C. Fernandes , “Wound Healing Treatment by High Frequency Ultrasound, Microcurrent, and Combined Therapy Modifies the Immune Response in Rats,” Brazilian Journal of Physical Therapy 20, no. 2 (2016): 133–141, 10.1590/bjpt-rbf.2014.0141. 26786082 PMC4900035

[25] N. X. Landén , D. Li , and M. Ståhle , “Transition From Inflammation to Proliferation: A Critical Step During Wound Healing,” Cellular and Molecular Life Sciences 73, no. 20 (2016): 3861–3885, 10.1007/s00018-016-2268-0. 27180275 PMC5021733

[26] H. E. Talbott , S. Mascharak , M. Griffin , D. C. Wan , and M. T. Longaker , “Wound Healing, Fibroblast Heterogeneity, and Fibrosis,” Cell Stem Cell 29, no. 8 (2022): 1161–1180, 10.1016/j.stem.2022.07.006. 35931028 PMC9357250

[27] H. Khalil , O. Kanisicak , R. J. Vagnozzi , et al., “Cell‐Specific Ablation of Hsp47 Defines the Collagen‐Producing Cells in the Injured Heart,” JCI Insight 4, no. 15 (2019): e128722, 10.1172/jci.insight.128722. 31393098 PMC6693833

[28] G. Sasaki , J. Grossman , and L. Misell , “Stimulation of Collagen Synthesis in Human Skin Following Ultherapy,” Poster of the 18th American Society for Dermatologic Surgery (ASDS) Annual Meeting, Schaumburg, IL, 2018, https://thebodywork‐clinic.co.uk/wp‐content/uploads/collagen‐synthesis‐stimulation‐with‐ultherapy.pdf.

[29] H. B. Xiao , R. H. Liu , G. H. Ling , et al., “HSP47 Regulates ECM Accumulation in Renal Proximal Tubular Cells Induced by TGF‐β1 Through ERK1/2 and JNK MAPK Pathways,” American Journal of Physiology. Renal Physiology 303, no. 5 (2012): F757–F765, 10.1152/ajprenal.00470.2011. 22718885 PMC3468491

[30] H. J. Laubach , I. R. Makin , P. G. Barthe , et al., “Intense Focused Ultrasound: Evaluation of a New Treatment Modality for Precise Microcoagulation Within the Skin,” Dermatologic Surgery 34, no. 5 (2008): 727–734, 10.1111/j.1524-4725.2008.34196.x. 18429926

[31] W. M. White , I. R. Makin , M. H. Slayton , et al., “Selective Transcutaneous Delivery of Energy to Porcine Soft Tissues Using Intense Ultrasound,” Lasers in Surgery and Medicine 40, no. 2 (2008): 67–75, 10.1002/lsm.20613. 18306156

[32] J. Y. Park , S. Youn , W. Hong , K. C. Lee , and I. Kim , “Treatment Protocol on Using Microfocused Ultrasound With Visualization for Skin Quality Improvement: The Korean Experience,” Plastic and Reconstructive Surgery. Global Open 11, no. 5 (2023): e5029, 10.1097/GOX.0000000000005029. 37250837 PMC10219723

[33] G. Casabona and N. Michalany , “Microfocused Ultrasound With Visualization and Fillers for Increased Neocollagenesis: Clinical and Histological Evaluation,” Dermatologic Surgery 40, no. Suppl 12 (2014): S194–S198, 10.1097/DSS.0000000000000231. 25417575

[34] W. P. Werschler and P. S. Werschler , “Long‐Term Efficacy of Micro‐Focused Ultrasound With Visualization for Lifting and Tightening Lax Facial and Neck Skin Using a Customized Vectoring Treatment Method,” Journal of Clinical and Aesthetic Dermatology 9, no. 2 (2016): 27–33. 27047630 PMC4771387

[35] Y. A. Yutskovskaya , A. D. Sergeeva , and E. A. Kogan , “Combination of Calcium Hydroxylapatite Diluted With Normal Saline and Microfocused Ultrasound With Visualization for Skin Tightening,” Journal of Drugs in Dermatology 19, no. 4 (2020): 405–411, 10.36849/JDD.2020.4625. 32272518

[36] G. Casabona , “Combined Calcium Hydroxylapatite Plus Microfocused Ultrasound for Treating Skin Laxity of the Chest and Buttocks,” Journal of Drugs in Dermatology 21, no. 1 (2022): 27–30, 10.36849/JDD.2022.6368. 35005869

[37] K. Darji , Parallel Ultrasound Beams Versus Microfocused Ultrasound for the Treatment of Upper Inner Arm Skin Laxity (Baltimore: ASLMS, Cadium, 2024). 10.1097/DSS.0000000000004881 41025649

